# Orexin A Inhibits Propofol-Induced Neurite Retraction by a Phospholipase D/Protein Kinase C_ε_-Dependent Mechanism in Neurons

**DOI:** 10.1371/journal.pone.0097129

**Published:** 2014-05-14

**Authors:** Karin Björnström, Dean Turina, Tobias Strid, Tommy Sundqvist, Christina Eintrei

**Affiliations:** 1 Department of Medical and Health Sciences, division of Anaesthesiology, Faculty of Health Sciences, Linköping University, Linköping, Sweden; 2 Department of Clinical and Experimental Medicine, division of Medical Microbiology, Faculty of Health Sciences, Linköping University, Linköping, Sweden; 3 Clinic of Anaesthesiology, Östergötland County Council UHL, Linköping, Sweden; Florida State University, United States of America

## Abstract

**Background:**

The intravenous anaesthetic propofol retracts neurites and reverses the transport of vesicles in rat cortical neurons. Orexin A (OA) is an endogenous neuropeptide regulating wakefulness and may counterbalance anaesthesia. We aim to investigate if OA interacts with anaesthetics by inhibition of the propofol-induced neurite retraction.

**Methods:**

In primary cortical cell cultures from newborn rats’ brains, live cell light microscopy was used to measure neurite retraction after propofol (2 µM) treatment with or without OA (10 nM) application. The intracellular signalling involved was tested using a protein kinase C (PKC) activator [phorbol 12-myristate 13-acetate (PMA)] and inhibitors of Rho-kinase (HA-1077), phospholipase D (PLD) [5-fluoro-2-indolyl des-chlorohalopemide (FIPI)], PKC (staurosporine), and a PKCε translocation inhibitor peptide. Changes in PKCε Ser^729^ phosphorylation were detected with Western blot.

**Results:**

The neurite retraction induced by propofol is blocked by Rho-kinase and PMA. OA blocks neurite retraction induced by propofol, and this inhibitory effect could be prevented by FIPI, staurosporine and PKCε translocation inhibitor peptide. OA increases via PLD and propofol decreases PKCε Ser^729^ phosphorylation, a crucial step in the activation of PKCε.

**Conclusions:**

Rho-kinase is essential for propofol-induced neurite retraction in cortical neuronal cells. Activation of PKC inhibits neurite retraction caused by propofol. OA blocks propofol-induced neurite retraction by a PLD/PKCε-mediated pathway, and PKCε maybe the key enzyme where the wakefulness and anaesthesia signal pathways converge.

## Introduction

General anaesthesia is a standard procedure for most surgery, used routinely on patients of all ages. The exact mechanism(s) on how anaesthesia is(are) achieved on the cellular level is not known but increasingly evidence shows that the crosstalk between different brain regions are reduced[1], [2]. In previous work, we have shown that the intravenous anaesthetic propofol causes reversible neurite retraction, leaving a thin threadlike structure behind, called a trailing remnant. Propofol also reverses the transport of neurite vesicles in rat cortical neurons by a γ-aminobutyric acid type A receptor (GABA_A_R)-mediated interaction with the cytoskeleton[3], [4]. When the neurites retract, the cell loses the precise contact to the adjacent cells, which in combination with the retrograde transport of vesicles away from the tip of the neurite might reduce the cell-cell communication. When propofol is omitted, the neurite extends again along the trailing remnant and re-establishes cell contact. The propofol signalling pathway includes modulation of the GABA_A_R, leading to the hyperpolarisation of the neuron[5] and an increase in intracellular calcium[6]. The effects on the cytoskeleton include phosphorylation of actin[7], redistribution of actin between cellular compartments dependent on rho/Rhokinase (ROK)[8], as well as morphological changes[9]. RhoA-kinase also interferes with propofol-induced rearrangement of cytoskeletal actin[8], and the retraction is also dependent on actomyosin contraction[3].

Recent data demonstrate that the induction and emergence paths through which anaesthetic-induced unconsciousness arise and dissipate are not identical [10]. The hypothalamic neuropeptide orexin-A (OA), involved in the control of sleep and wakefulness, is also linked to emergence from general anaesthesia [11], [12]. Intracerebroventricular administration of OA reduces the anaesthetic effect of several intravenous and volatile anaesthetics[13]–[15] in rats. Orexin A producing neurons project throughout the central nervous system (CNS) to regulate the sleep-wake cycle, as well as autonomic and neuroendocrine functions[16], [17]. OA could be a valuable tool to understand the anaesthetic mechanisms. OA interacts with two G(q)-coupled receptors, orexin_1_ and orexin_2_ (OXR_1_ and OXR_2_)[16], [17], inducing a rise of intracellular calcium[16], activation of phospholipase C and D (PLC and PLD)[18] that produces phosphatidic acid (PA) and choline. PA is further metabolised to lysophosphatidic acid (LPA) and diacylglycerol (DAG)[19]. DAG activates PKC and facilitates the translocation of PKC from the cytosol to the plasma membrane[20].

The aim of this study is to investigate whether OA interferes with neurite retraction induced by the intravenous anaesthetic propofol in cultured rat brain cells and its signalling pathway.

## Methods

### Cell culture

The study was approved by the Linköping Ethics Committee for Animal Research, Dnr 113/11. Primary cultures of mixed rat neurons/glial cells were obtained essentially as described by Hansson and Rönnbäck[21] and modified according to Björnström[6]. The cells were grown on poly-L-lysine coated cell flasks or glass cover-slips and used on day 12–30 when they showed matured morphology[21], [22], with no differences in cellular response.

### Live cell microscopy

The coverslip was rinsed twice in calcium-containing medium (CCM) and mounted in a closed bath imaging chamber placed in a heated stage to reach 37°C. Cells were observed by light microscopy (Zeiss Axiovert 135 M (Carl Zeiss Gmbh, Göttingen, Germany) with a 40x [numeric aperture 1.3] oil immersion objective). Differential interference contrast images of cells were taken, processed and stored as previously described[3]. We analysed only superficial cells with a neuronal morphology, *i.e* with at least one long cellular protrusion (a neurite) - most often with vesicles in the protrusion, lying on a glial cell layer. The neurite had to be visible for the entire experiment. Time-lapse series were obtained at 1 min intervals, with application of drugs 15 sec before the measurement image was captured. Neurite length was measured manually[3] from those images using Adobe PhotoShop 6.0 (Adobe Systems, San Jose, CA) and ImageJ (Rasband, W.S., ImageJ, U.S. National Institutes of Health, Bethesda, MD, USA, http://rsb.info.nih.gov/ij/, 1997–2005). Measurement obtained from a single neurite is defined as (n = 1) and the neurons were obtained from at least 3 different rat litters in each group. The length of the neurite at time (-1) was used as the reference point (100%). After the experiment, the area around the oil drop was marked on some coverslips used for live cell imaging. To identify neuronal cells, they were immunolabelled for β_3_ tubulin. The cover-slips were fixed for 30 min in 4% paraformaldehyde in phosphate buffered saline (PBS), rinsed, and mouse anti-tubulin-β_3_ antibody (1∶500, Thermo Fisher scientific, Waltham, MA, USA) followed by Alexa-546-conjugated antibody (1∶400, Invitrogen, Paisley, UK). All antibodies were diluted in 1% bovine serum albumine (BSA)/0.1% saponin/PBS and incubated for 45 min, rinsed and mounted on object glass. Thereafter the cell used in the live-cell imaging was identified with a 63x oil- fluorescence objective, numeric aperture (NA) 1.4 (Axiovert 200 M, Carl Zeiss, Göttingen, Germany) equipped for DIC light microscopy, and thereafter evaluated for fluorescence.

**Figure 1:**
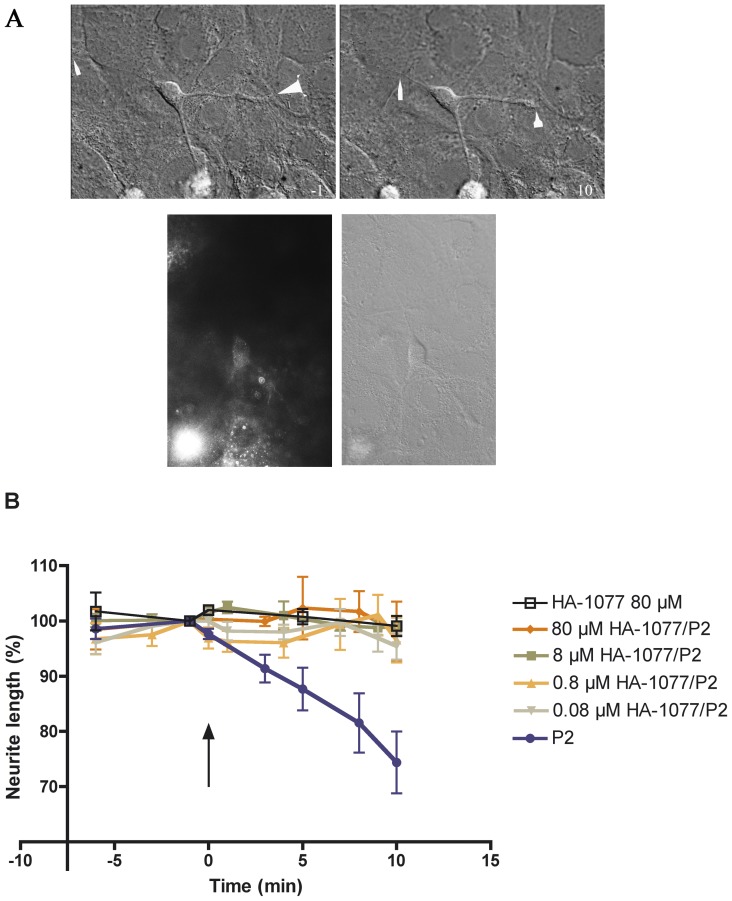
(A): Time-lapse imaging reveals the dynamics of neurite retraction after addition of propofol. Upper panel: Cortical cell cultures were treated with CCM for 5 min (-5 to 0 min), exposed to 2 µM propofol and observed for 10 min. Images shown were taken -1 and 10 min following addition of propofol. The arrows indicate the tip of the neurites, with the neurite extending towards the upper left corner show a trailing remnant (very thin treadlike structure). Lower panel: The same cell identified with DIC microscopy (right) after fixation with 4% PFA/PBS for 30 min, followed by immunostaining of β_3_ tubulin to identify neuronal cells (left). The neurite with the trailing remnant is out of focus in the fluorescent picture. Cell orientation is different, as the cell is examined in different microscopes for the upper and lower panels. (B): Propofol-induced neurite retraction is dependent on Rho Kinase. Graph of time-dependent response of cortical cell cultures in CCM that were pretreated with the HA-1077 0.08-80 µM for 40 min, observed for 5 min in CCM-HA1077 and then exposed to 2 µM propofol (P2) for 10 min. Propofol addition is shown by an arrow. Values are expressed as percentage of neurite length (100%) 1 min before propofol addition and represent mean ± SEM. Data were based on at least 5 neurites in each HA-1077/propofol group and n = 9 cells, 10 neurites in the propofol group. Propofol induced a neurite retraction to 74.4±5.6% of initial length. Pretreatment of the cells with the RhoA-kinase inhibitor HA-1077 (0.08 – 80 µM) for 40 min blocked the propofol-induced neurite retraction to (95.5±2.5%, n = 6) for 0.08 µM after 10 min, with the same blocking effect for 0.08 – 80 µM HA-1077, (n = 5 each). All concentations tested were significantly different from propofol after 5 min and onwards (p<0.001, 2-way ANOVA with Bonferroni post-hoc test). No retraction was seen by 80 µM HA-1077 alone (99.1±1.8%, n = 5).

Neurite length was measured after CCM for 5 min to establish the steady state, followed by propofol (2 µM) administration (Figure 1). Each cover-glass was used for only one treatment, but two cells could be used for evaluation if they were within the same view-field. Cells that for any reason were spontaneously retracting were discarded. ROK was inhibited by 1-5(-isoquinolinesulfonyl) homopiperazine (HA-1077)[23], also known as fasudil (Sigma Chemical Co., St. Louis, MO, USA). Cells were incubated with HA-1077 (0.08 - 80 µM) in cell culturing media for 40 min prior to incubation with CCM/HA-1077 (5 min) to establish the steady state. Thereafter, commercial propofol (2 µM, dissolved in the lipid solution Lipuro; both from Braun, Melsungen, Germany) was added and the neurite was followed for a further 10 min. The lipid vehicle does not interfere with neurite retraction[3], [8]. To study the OA effects of propofol, OA (10 nM) was added 1 min before propofol (2 µM). To inhibit PLD, 5-fluoro-2-indolyl des-chlorohalopmide[24] (FIPI, 100 nM, Sigma Co.) was added to cell culturing media for 60 min prior to steady state measurement in CCM/FIPI (5 min). Thereafter, OA (10 nM) was added for 1 min before propofol (2 µM) and the neurite was followed for a further 15 min. In PKC experiments, the cells were incubated with CCM (5 min) to establish the steady state. Thereafter, staurosporine[25] (3 nM) was added for 5 min. This was followed by the addition of OA (10 nM) or the solvent for OA (acetic acid [AE, 0.001%]) for 1 min followed by propofol (2 µM) for 10 min. To activate PKC, 100 nM phorbol 12-myristate 13-acetate (PMA) was added to the cells 3 min prior to propofol addition (2 µM) for further 15 min. The PKCε translocation inhibitor peptide[26] (PKCεI) (5 µM; Calbiochem, Merck Millipore, Darmstadt, Germany) was added in cell culture media for 45 min, followed by 5 min in CCM before addition of OA (10 nM, 1 min) and thereafter propofol (2 µM) for 10 min. For control experiments, the inhibitors alone continued for the total time of the experiment.

A limitation of our study was that our cultures consisted of mixed neuron-glial cells, and we analysed only superficial cells lying on a glial cell layer. The findings in this study were obtained from *in vitro* experiments and the cells in time-lapse experiments were chosen based on our judgment that their morphology resembled that of neurons. This is a subjective choice, and we can only confirm that they were β_3_ -tubulin-positive afterwards.

### Analysis of cellular proteins

Cells in 25-cm^2^ culture flasks were washed twice in CCM, then incubated with either CCM, OA (10 nM) or propofol (2 µM) for 10 minutes in CCM at 37°C in a waterbath. The CCM and propofol treated flasks received AE 0.001% (added 1 min before propofol). The effect of PLD was assessed by pretreatment for 1 h with FIPI (100 nM) in cell culture media, followed by washing twice in CCM-FIPI. The experiment was thereafter done as described above, with drugs added to CCM-FIPI. After removal of stimulation medium, ice-cold lysis buffer with phosphatase inhibitors (250 µl) was added and subsequent procedures were carried out at 4°C or on ice[6]. Cells were scraped off into the lysis buffer, homogenised and the cell lysate centrifuged (2×10 min, 200 *g*) to remove remaining intact cells and nuclei. Protein concentration was measured by spectrophotometry and samples were diluted with the lysis buffer to equal relative protein concentration in each experiment. 70 µl of lysate was mixed with sample buffer[27], followed by heating for 15 minutes at 65°C and thereafter frozen until analysed. Frozen samples were heated at 95°C for 10 min and then separated on homogenous 8% polyacrylamid gels in the presence of sodium dodecyl sulphate (SDS)[6]. Proteins in the gel were blotted to polyvinyldifluoride (PVDF) membrane blocked in 2% BSA/PBS, incubated with PKCε-phospho-Ser^729^ antibodies (Abcam PLC, Cambridge, UK) (1∶1000 in 1% PBS-BSA) for (2 h (rt) or overnight (4°C)). The membrane was washed six times (PBS-Tween 0.05 %) and incubated with peroxidase-linked goat anti-rabbit antibodies (1∶5000 in 0.05% PBS-Tween, 1 h, rt). After extensive washing in PBS-Tween, the membrane was incubated with enhanced chemiluminiscence (ECL) Western blotting detection reagents and visualized using a chemiluminiscence sensitive camera.

### Statistical analysis

Overall significant differences between conditions were determined by two-way analysis of variance (ANOVA) with repeated measures. Post hoc comparisons were performed using the Bonferroni test for multiple comparisons. A p value of <0.05 was considered statistically significant. The values were expressed as the mean ± standard error of the mean (SEM). All statistical analyses and graphing were carried out using Prism 4.0 software (GraphPad Software, San Diego).

## Results

### Propofol induces neurite retraction through a RhoA-kinase-dependent mechanism

The anaesthetic propofol (2 µM) caused a time-dependent neurite retraction to 74.4±5.6% of the initial value (n = 10) after 10 min of stimulation (Fig. 1A and 1B). To explore the signal cascade of how propofol caused retraction, we tested whether activation of RhoA-kinase, known to interfere with propofol-induced rearrangement of cytoskeletal actin, was involved. Pretreatment of the cells with the RhoA-kinase inhibitor HA-1077 (80 µM) for 40 min blocked the propofol-induced neurite retraction after 10 min, 98.1±5.4%, n = 6, p<0.01, with the same blocking effect for HA-1077 (0.08 – 8 µM, n = 5 each), and significantly different compared with propofol after 5 min and onwards (p<0.001) for all concentrations. HA-1077 at 80 µM alone had no effect on neurite length (99.1±1.8%, n = 5).

### Orexin A inhibits propofol-induced neurite retraction by activation of phospholipase D

**Figure 2:**
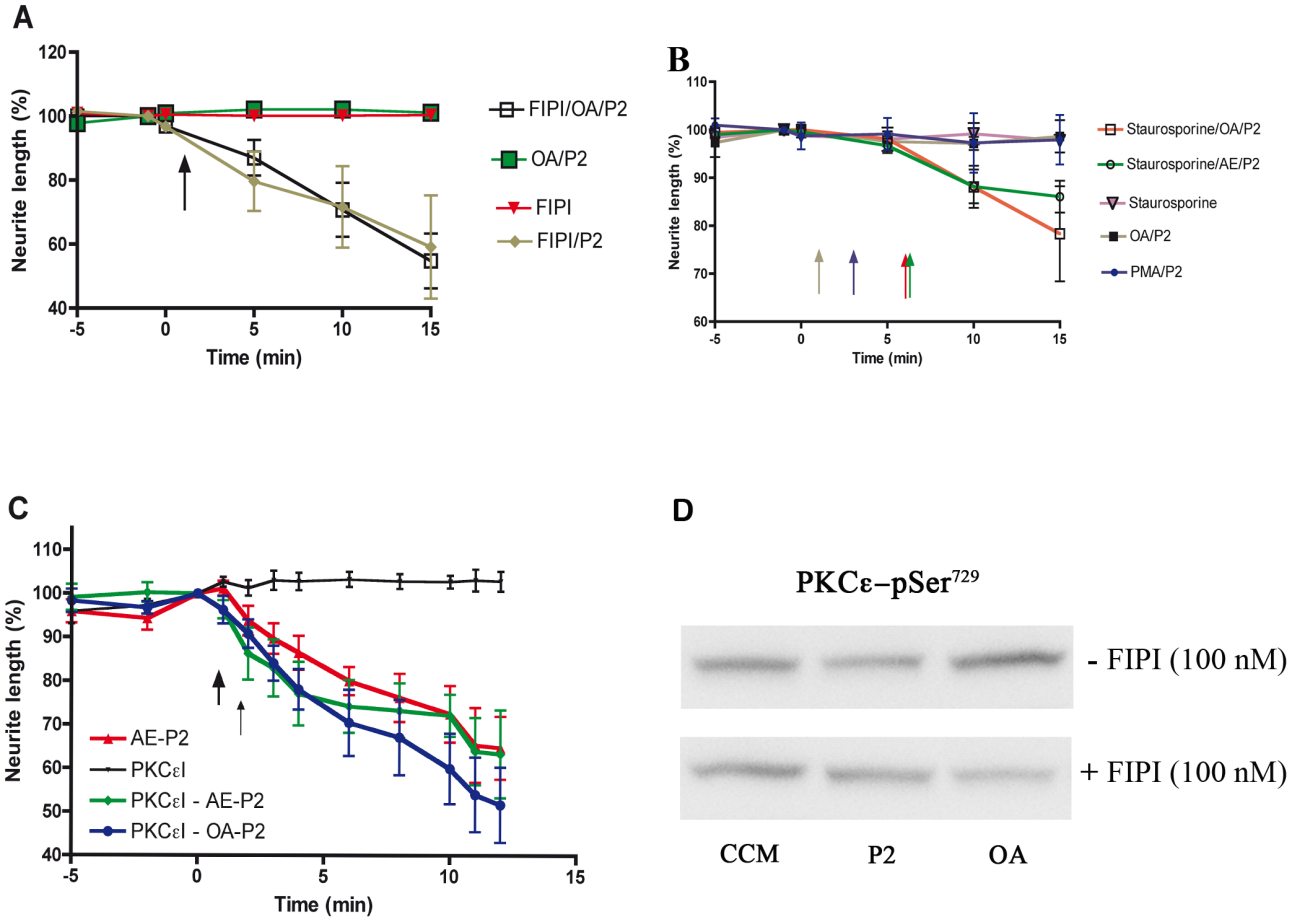
(A): Orexin A inhibits propofol-induced neurite retraction by activation of phospholipase D. Graph of time-dependent response of cortical cell cultures in CCM pretreated with the PLD inhibitor FIPI (100 nM) for 60 min, observed for 5 min in CCM-FIPI and exposed to 2 µM propofol (P2, arrow) for 15 min. OA (10 nM) was added 1 min before propofol exposure. Values are expressed as percentage of neurite length (100%) 1 min before OA addition and represent mean ± SEM. The propofol-induced retraction was blocked with OA (101.1±2.2%, n = 6). FIPI prevented the inhibitory effect of OA on propofol-induced neurite retraction already after 5 min and caused retraction to (54.7±8.6%, n = 6), after 15 min. No retraction was seen by FIPI alone (100.4±0.5%, n = 6). Propofol retraction is not inhibited by FIPI (59.1± 16.1%, n = 3) at 15 min. The retraction response for FIPI/P2 and FIPI/OA/P2 was significant from 5 min (p<0.001, 2-way ANOVA followed by Bonferroni post-hoc test). (B) The inhibitory effect of Orexin A on propofol-induced neurite retraction is protein kinase C-dependent. Graph of time-dependent response of cortical cell cultures first observed for 5 min in CCM, and thereafter pretreated with the PKC inhibitor staurosporine (3 nM) for 5 min and exposed to 2 µM propofol (P2) for 10 min. OA (10 nM) or the OA solvent acetic acid (AE, 0.001%) was added 1 min before propofol exposure. Values are expressed as percentage of neurite length (100%) 1 min before OA/AE addition and represent mean ± SEM. OA block the propofol-induced retraction (98.6±3.4%, n = 6). No retraction was seen by staurosporine alone (97.8±0.9, n = 6). Staurosporine prevented the inhibitory effect of OA on propofol-induced neurite retraction (78.3±9.9%, n = 7), 10 min after propofol addition, p<0.001 compared with OA/P2 (2-way ANOVA, followed by Bonferroni post-hoc test). Staurosporine did not affect the response of AE/P2 after 10 min (neurite retraction (86.1±3.3%, n = 7, p<0.05 compared with OA/P2). Pretreatment with the PKC activator PMA (100 nM) for 3 min abolished the propofol-induced neurite retraction after 15 min (97.9±5.2 %, n = 6). The colour-coded arrow indicates propofol addition for each experiment. (C) The orexin effect is due to translocation of protein kinase Cε. Graph of time-dependent neurite retraction on cortical cell cultures pre-incubated for 45 min with the PKCε translocation inhibitor peptide (PKCεI, 5 µM), stimulated with OA (10 nM) or the OA solvent acetic acid (AE, 0.001%) 1 min (thick arrow) before propofol (2 µM (P2), thin arrow) exposure for 11 min, the PKCεI alone or AE/P2. PKCεI alone did not change neurite length (102.8 ±2.3%, n = 5). AE/P2 retracted the neurite to (64.7±7.2%, n = 4), non significant compared with PKCεI/AE/propofol (63.3±10.0%, n = 7, 2-way ANOVA, followed by Bonferroni post-hoc test). When PKCε cannot translocate from the cytosol to the membrane, OA could not prevent retraction (51.6±8.6%, n = 10) at 10 min after propofol addition. All propofol treatments were significantly different from PKCεI (p<0.001). (D) Orexin A activates PKCε via a PLD dependent phosphorylation of PKCε Ser^729^ whereas propofol reduces PKCε Ser^729^ phosphorylation. Western blot analysis of PKCε Ser^729^ phosphorylation on cortical cell cultures treated with CCM, P2 (2 µM, 10 min), or OA (10 nM, 11 min), with or without FIPI (100 nM). CCM and P2 cells were treated with acetic acid (0.001%) for 11 min (CCM) or 1 min before addition of propofol (P2). FIPI was preincubated for 1 h, and supplemented throughout the experiment. Blots were visualized with an anti-PKCε Ser^729^ phosphorylation antibody (1∶1000)/horseradish peroxidase linked anti-rabbit antibody (1∶5000). OA increases the PKCε Ser^729^ phosphorylation compared to CCM, and this is reduced when PLD is blocked by FIPI, whereas propofol-treated cells showed a decrease in PKCε Ser^729^ phosphorylation that increased after FIPI treatment (n = 5). The lanes shown are from the same blot, but rearranged into rows.

OA (10 nM), the regulator of wakefulness, was added 1 min before the anaesthetic propofol to evaluate if OA could interfere with propofol. The propofol-induced retraction was blocked (101.1±2.2% of initial neurite length, n = 6). The signal cascade of OA includes PLD and PKC. Pretreatment of the cells with a PLD inhibitor (FIPI, 100 nM) prevented the inhibitory effect of OA on the propofol-induced retraction of the neurites, allowing propofol to retract the neurite length to (54.7±8.6%, n = 6) at 15 min (Fig. 2A). Propofol retraction is not inhibited by FIPI (59.1± 16.1%, n = 3, non-significant compared with FIPI/OA/P2). The retraction response for FIPI/P2 as well as FIPI/OA/P2 was significantly different (p<0.001) from OA/P2 at 5 min and onwards. No retraction was seen by FIPI alone (100.4±0.5%, n = 6).

### Protein kinase C is important for the inhibitory effect of Orexin A on propofol-induced neurite retraction

In the signalling cascade of OA, PLD activates PKC, which induce phosphorylation of several proteins. In this experiment, OA (10 nM) added 1 min before propofol, blocked the propofol-induced retraction (98.6±3.4%, n = 6). The PKC inhibitor staurosporine, 3 nM, prevented this inhibitory effect of OA on the propofol-induced retraction of the neurites after 10 min, and thus the neurite length was 78.3±9.9%, n = 7, p<0.001. Staurosporine on its own had no effect on the neurite length (97.8±0.9, n = 6) and did not affect the response of AE/P2 after 10 min of propofol treatment (neurite retraction (86.1±3.3%, n = 7, p<0.05 compared with OA/P2). Activation of PKC by PMA (100 nM) inhibits propofol-induced neurite retraction, where a brief 3 min pretreatment abolished the retraction after 15 min (97.9±5.2 %, n = 6, Fig. 2B).

### The orexin effect is due to activation of protein kinase Cε

Staurosporine is a pan-PKC blocker, so the results obtained with straurosporine might be skewed. Activation of PKCs is needed to move the enzyme from the cytosol to the cellular membrane. PKCε has an actin-binding motif, making it a possible candidate for regulating retraction. By blocking the translocation of PKCε to subcellular sites with a blocking peptide (PKCεI), this PKC is not available for the signal cascade. No effect was seen with PKCεI alone (102.8 ±2.3%, n = 5). When the translocation inhibitor peptide was pre-incubated for 45 min before addition of OA/propofol, the cell retracted (51.6±8.6%, n = 10, p<0.001 compared with PKCεI). Cells treated with the translocation inhibitor and propofol, retracted (63.3±10.0%, n = 7), similar to propofol-treated cells (64.7±7.2%, n = 4, Fig. 2C).

When PKCε is activated, it becomes phosphorylated upon Ser^729^. Western blot analysis showed that OA increases the PKCε Ser^729^ phosphorylation, whereas propofol reduced it compared with CCM, n = 5. When FIPI was used to block PLD, OA reduced PKCε Ser^729^ phosphorylation. Propofol treated cells showed a higher degree of phosphorylation upon PKCε Ser^729^ after PLD inhibition (Fig. 2D).

## Discussion

The intravenous anaesthetic propofol induced reversible neurite retraction[3] that changed the morphology of the cell and reduced its cellular contact to adjacent cells. In this study we showed that this retraction was blocked by HA-1077, a selective ROK inhibitor. The neuropeptide OA, involved in regulating awakeness, inhibited this retraction through the activation of PLD and PKCε by changing the phosphorylation of a crucial amino-acid of PKCε that activates the enzyme and translocates it from the cytosol to the cell membrane[28]. A PKC activator, PMA, also inhibited propofol-induced neurite retraction.

The signal cascade used by propofol, to cause changes in cytoskeletal actin organisation and retraction of the neurites, involve the GABA_A_ receptor (GABA_A_R), RhoA, ROK, an increase in intracellular calcium concentration[6]–[8] and include activation of actin-myosin-dependent contraction[3]. The downstream effector of RhoA, ROK, has been shown to phosphorylate myosin light-chain[29]. This, in turn, enhances the binding of myosin to actin filaments, contributing to neurite retraction[30]. We have shown previously that blebbistatin, a myosin II ATPase inhibitor, and phalloidin, an F-actin stabilising agent, also block propofol-induced neurite retraction[3]. Actin is also changed in cellular distribution after propofol stimulation; this process is dependent on RhoA and ROK. Previous work has shown that the ROK inhibitor used in this study, HA-1077, protects cultured neuroblastoma cells against lysophosphatidic acid (LPA)-induced neurite retraction[31]. This is consistent with our results (Fig. 1), showing that HA-1077 blocks propofol-induced neurite retraction in cultured cells. Taking our previous results together with data in Fig. 1 it implicates a possible signalling pathway for propofol-induced neurite retraction that involves RhoA/ROK, causing myosin light-chain (MLC) phosphorylation followed by actin and myosin contractility that will retract the neurite.

OA is a neuropeptide that regulates wakefulness, but it has also been implicated in reducing anaesthetic effects[13]–[15] and might be a tool to understand anaesthetic mechanisms. We show that 10 nM OA could prevent neurite retraction (Fig. 2A). OA binding to OXR causes PLD activation followed by DAG production[18], which overlaps with PKC activation[32]. An increase in DAG causes PKC activation and its translocation to the membrane[33]. We could block the inhibitory effect of OA on propofol-induced neurite retraction (Figs. 2A and 2B) using the PLD inhibitor FIPI and the PKC inhibitor staurosporine, confirming the importance of PLD for OA signalling.

The PKC family consists of 10 different isoforms[34], including classical members (α, β, γ) whose activation requires both Ca^2+^ and DAG, novel PKCs (δ, ε, η, θ) activated only by DAG and atypical PKCs (ι, τ, λ), whose activation is not dependent on Ca^2+^ or DAG. PKCε are abundant in the nervous system and promote neurite outgrowth[35] via its interaction with the actin binding motif with actin filaments. Overexpression of PKCε induces neurite outgrowth in neural cells via its regulatory domain[36] and by suppression of RhoA activity[37]. When PKCε is activated on its kinase domain, it becomes phosphorylated upon Ser^729^, which makes the catalytic site more active. This is followed by translocation of the PKCε to the cell membrane and activation of the actin-binding motif[28]. By blocking the translocation of PKCε, OA could no longer block the neurite retraction caused by propofol (Fig. 2C). OA also increases the phosphorylation of PKCε Ser^729^, and this phosphorylation is markedly reduced when PLD is blocked by FIPI (Fig. 2D). Propofol instead showed a decrease of PKCε
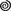
Ser^729^ compared with unstimulated cells. This is contrast to previous data, where propofol increased phosphorylation of PKCε Ser^729^ in dorsal root ganglions[38], causing PKCε Ser^729^ to translocate to the membrane. We did not see any effect on the propofol-induced retraction when translocation was inhibited by PKCε I (Fig. 2C). Our data suggest that propofol counter balance the normal activity of cellular PKCε; the signalling pathway might include PLD as inhibition of PLD restores PKCε Ser^729^ phosphorylation (Fig. 2D). Propofol could interfere with the PLD/PKC pathway via RhoA, as there is evidence for direct interactions between PKC and RhoA, and these interactions could result in significant cross-talk between the pathways regulated by RhoA and PKC via PLD[39], [40]. This suggests that the anaesthetic and wakefullness signalling pathways interfere at a single point.

**Figure 3:**
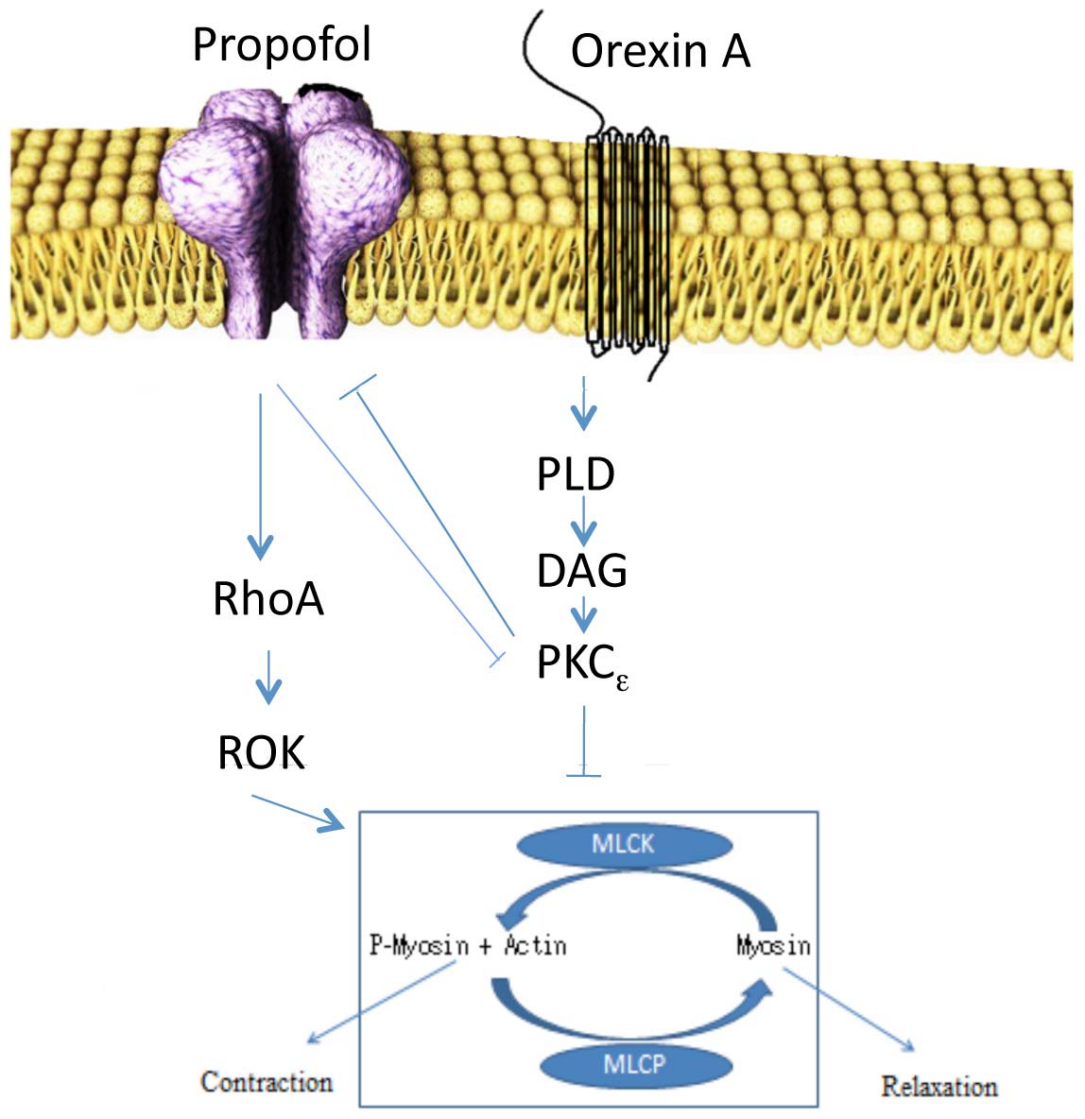
Proposed pathway for Orexin A inhibition of propofol-induced neurite retraction. A proposed schematic view of how propofol and OA interfere with neurite retraction. Propofol binds to GABA_A_R and causes neurite retraction through the RhoA/ROK pathway by activating the acto-myosin complex (blue box), where phosphorylation of myosin via myosin light chain kinase (MLCK)[3] causes contraction of the neurite. When myosin is de-phosphorylated via myosin light chain phosphatase (MLCP) the neurite extends. OA binds to OXR and activates PLD, increasing DAG, which activates PKCε by increasing the phosphorylation of PKCε Ser^729^. The activated PKCε translocates from the cytosol to the cell membrane. The now activated PKCε then interfere with a membrane effector, possibly the GABA_A_R[43], which might cause a decrease in the amount of GABA_A_R at the cell surface. PKCε also have an actin binding motif, that could directly interfere with the cytoskeletal actin involved in the contractile response causing neurite retraction. PKCε stabilizes F-actin[28] when bound, and then retraction could not take place. Propofol reduces the phosphorylation of PKCε Ser^729^ below the amount in unstimulated cells, suggesting that propofol counter-balances the normal activity of cellular PKCε; the signalling pathway of propofol might include PLD as inhibition of PLD restore PKCε Ser^729^ phosphorylation. The exact pathway used, is yet to be determined.

The intracellular signalling used by OA to block neurite retraction caused by propofol is not fully understood. Our data suggest a PLD/PKCε Ser^729^ phosphorylation, where the activation of PKCε Ser^729^ is crucial, as both propofol and OA changed the phosphorylation in opposite ways. For OA, translocation of activated PKCε to the cell membrane is important, where it may interact with a target structure as a kinase. It has been shown that PKCε reduces the sensitivity of GABA_A_R to barbiturates, benzodiazepines, neurosteroids and ethanol[41], [42], and this is most likely the case for propofol. PKCε decreases the amount of GABA_A_R at the cell surface and attenuates GABA_A_R currents[43]. In cortical neurons, PKC inhibitors abolish phosphorylation of the GABA_A_R β_3_ subunit and increase receptor activity, whereas activators of PKC enhance β_3_ phosphorylation, leading to a decrease in channel activity[44]. We were able to inhibit propofol-induced neurite retraction by PMA, a potent PKC activator. This could indicate involvement of PKC in GABA_A_R modulation of sensitivity to propofol, and is a possible pathway by which OA could prevent propofol-induced neurite retraction. However, the activated PKCε also expose its actin-binding motif, where it helps stabilising F-actin[28]. Actin is a necessary part of the actomyosin-mediated contractility caused by propofol[3], and we have previously shown that propofol increased membranous actin in a rhoA/ROK-dependent way[8]. When F-actin is stabilized with phalloidin, propofol cannot cause retraction[3]. When PKCε is translocated to the membrane by OA, it could prevent the propofol-induced, rhoA/ROK-dependent turnover of actin and thereby stop the retraction process. A suggested signal pathway is described in Figure 3.

Taken together, at least two options for the mechanism of the OA inhibition of propofol-induced neurite retraction could be considered: One is a signalling pathway by which OA inducing PLD/PKC activation might lead to the reduction of GABA_A_R sensitivity to propofol and a decrease in the amount of GABA_A_R at the cell surface, which would in turn inhibit activation of the RhoA/ROK/MLC pathways. The other possibility is that PKC activation interferes directly with actomyosin-mediated contractility. The dominant pathway is still to be determined.

In conclusion, the results from our study suggest that the RhoA-ROK- signalling pathway has an essential role in the regulation of propofol-induced neurite retraction, most probably by interaction with the actomyosin complex. The results further indicated that PLD-PKCε activation is important for the OA inhibition of propofol-induced neurite retraction, via increased phosphorylation of PKCε Ser^729^ and translocation of the enzyme.
